# Response to Intravenous N-Acetylcysteine Supplementation in Critically Ill Patients with COVID-19

**DOI:** 10.3390/nu15092235

**Published:** 2023-05-08

**Authors:** Yenifer Gamarra-Morales, Lourdes Herrera-Quintana, Jorge Molina-López, Héctor Vázquez-Lorente, Juan Francisco Machado-Casas, José Castaño-Pérez, José Miguel Pérez-Villares, Elena Planells

**Affiliations:** 1 Clinical Analysis Unit, Valle de los Pedroches Hospital, Pozoblanco, 14400 Córdoba, Spain; 2 Department of Physiology, School of Pharmacy, Institute of Nutrition and Food Technology “José Mataix”, University of Granada, 18071 Granada, Spain; 3 Faculty of Education, Psychology and Sports Sciences, University of Huelva, 21007 Huelva, Spain; 4 Intensive Care Unit, Virgen de las Nieves Hospital, Fuerzas Armadas Avenue, 18014 Granada, Spain

**Keywords:** COVID-19, N-acetylcysteine, mortality, antioxidant, pneumonia, biomarker

## Abstract

Administering N-acetylcysteine (NAC) could counteract the effect of free radicals, improving the clinical evolution of patients admitted to the Intensive Care Unit (ICU). This study aimed to investigate the clinical and biochemical effects of administering NAC to critically ill patients with COVID-19. A randomized controlled clinical trial was conducted on ICU patients (*n =* 140) with COVID-19 and divided into two groups: patients treated with NAC (NAC-treated group) and patients without NAC treatment (control group). NAC was administered as a continuous infusion with a loading dose and a maintenance dose during the study period (from admission until the third day of ICU stay). NAC-treated patients showed higher PaO_2_/FiO_2_ (*p ≤* 0.014) after 3 days in ICU than their control group counterparts. Moreover, C-reactive protein (*p ≤* 0.001), D-dimer (*p ≤* 0.042), and lactate dehydrogenase (*p ≤* 0.001) levels decreased on the third day in NAC-treated patients. Glutathione concentrations decreased in both NAC-treated (*p ≤* 0.004) and control (*p ≤* 0.047) groups after 3 days in ICU; whereas glutathione peroxidase did not change during the ICU stay. The administration of NAC manages to improve the clinical and analytical response of seriously ill patients with COVID-19 compared to the control group. NAC is able to stop the decrease in glutathione concentrations.

## 1. Introduction

Severe acute respiratory syndrome coronavirus 2 (SARS-CoV-2) infection can cause dyspnea that can lead to acute respiratory distress syndrome (ARDS) leading to the production of a set of immune mediators against the invading virus [1], and a profile of unbalanced chemokines [2,3,4]. In this process, excessive free radicals are formed that cannot be counteracted by biological antioxidant systems [5]. These free radicals can negatively amplify the inflammatory response, producing cell damage (membrane, proteins, and DNA), and leading to cell dysfunction with or without disseminated intravascular coagulation, fulminant myocarditis [6], multi-organ failure [7,8], renal and hepatic failure and pneumothorax [9], and the possible death of the patient.

Glutathione plays a fundamental role in many biological processes essential for the homeostasis of the organism [10]. Glutathione in its reduced form (GSH) has a redox action that eliminates toxic peroxides produced during metabolism under aerobic conditions. The conversion of GSH to the oxidized form of glutathione (GSSG) is catalyzed by glutathione peroxidase (GPx). The oral and intravenous administration of glutathione has been studied in patients with ARDS secondary to COVID-19 pneumonia because it improves dyspnea a few hours after its administration [11]. The current literature suggests that glutathione deficiency would be the most plausible explanation for the severe manifestations and deaths in patients with COVID-19 [12].

N-acetylcysteine (NAC) might be beneficial for treating patients with COVID-19 because it helps restore glutathione levels, intervening in its synthesis. In addition, NAC has an antioxidant and anti-inflammatory effect and regulates the immune response. A high dose of intravenous NAC can be expected to play an adjunctive role in treating severe cases of COVID-19 and managing its lethal complications, including pulmonary and cardiovascular adverse events [13]. GSH is a metabolite that decreases with age [14] and in diseases such as diabetes mellitus and cardiovascular disease [15], decreases more in men than in women [16].

Several studies showed that the increase in neutrophils and neutrophil extracellular traps (NETs) in COVID-19 patients contribute to increasing severity and mortality. Therefore, they can be used as therapeutic targets [17]. Furthermore, NAC has been shown to inhibit NET formation by human neutrophils in vitro [18]. Moreover, NAC has been shown to prevent T-cell immunosuppression in a pro-oxidative environment [19] and thus can reverse lymphopenia in COVID-19.

The intervention with NAC was used successfully in patients with invasive mechanical ventilation, observing a decrease in ferritin and C-reactive protein (CRP) [20]. In addition, a clinical improvement and a decrease in several inflammatory markers (CRP, ferritin, and lactic acid) were found in a patient with multiple organ failure who received combined treatment with hydroxychloroquine and NAC (22). In addition, administering an inhaled NAC solution to patients with COVID-19 with unfavorable evolution after radical treatment of esophageal cancer and encapsulated right pneumothorax achieved progressive improvement and hospital discharge [21]. Finally, a phase I clinical trial in which a combination of methylene blue, vitamin C, and NAC was administered to COVID-19 patients admitted to the Intensive Care Unit (ICU) showed an adequate response, and they could be discharged from ICU [22].

Based on the information mentioned above, the present study proposes that the administration of NAC could counteract the effect of these free radicals, improving the antioxidant status and inflammatory situation and, therefore, the clinical evolution of the COVID-19 patient in the ICU. The main objective of our study was to investigate the clinical and biochemical effects of administering NAC to critically ill patients with COVID-19.

## 2. Materials and Methods

### 2.1. Patients and Study Design

A randomized, controlled clinical trial was conducted on critically ill patients with COVID-19. The design was a prospective, analytical, follow-up study of cases and controls. The sample of patients studied was made up of 140 consecutive patients over 18 years of age (women, 23.6%) admitted to the ICU with COVID-19. The groups’ distribution comprised a total of 72 patients treated with NAC (treated patients), and 68 patients not treated with NAC (control group patients). The sample size we used in our study is similar to the sample size of other studies similar to ours [23,24]. Patients were recruited from 1 March to 1 June 2020 after being informed about the study protocol which was signed by all the patients or the family. On admission (first day) and on the follow-up (third day) at Virgen de las Nieves Hospital in Granada (Spain) ICU, samples and analytical data were taken. All patients had a positive diagnosis of critical active SARS-CoV-2 infection (analyzed by Real-Time Reverse Transcriptase–PCR (RT-PCR)) testing of nasal and pharyngeal swab samples. Patients were considered critically ill when they presented respiratory failure requiring mechanical ventilation, needed vasopressor treatment (shock), or presented other complications with organ failure requiring monitoring or treatment in the ICU. Inclusion criteria were: (I) to be aged 18 years or older, (II) to be previously hospitalized for at least more than 48 h, (III) to be admitted to the ICU and to stay for at least 3 days, and (iv) to present a positive PCR test for SARS-CoV-2 according to the Chinese Clinical Guideline for the classification of COVID-19 [25]. The present study was conducted in accordance with the principles of the Declaration of Helsinki (last revised guidelines from 2013) [26], following the International Conference on Harmonization (ICH)/Good Clinical Practice (GCP) standards, and was approved by the Ethics Committee of the University of Granada (Ref. 149/CEIH/2016).

### 2.2. Treatment and Nutritional Support

Patients received treatment that included medications (antivirals, antibacterial, corticosteroids, etc.), respiratory support, and nutritional support (enteral, parenteral, and/or mixed enteral/parenteral) during the hospital stay. The latter was according to the Clinical Nutrition Units Guidelines of the hospitals, based on the American Society for Parenteral and Enteral Nutrition (ASPEN) and the European Society of Parenteral and Enteral Nutrition (ESPEN) guidelines [27]. The enteral nutrition provided to the study patients consisted of commercial formulas fed orally or tube fed for at least 3 days, providing >10 kcal/kg/d of energy. Parenteral nutrition consisted of administering at least 2 energy-providing nutrients, including glucose, fat emulsion, and amino acids, for at least 3 days, providing >10 kcal/kg/d of energy. Caloric administration during the early phase was hypocaloric, without exceeding 70% of energy expenditure as recommended by the ESPEN [28].

### 2.3. NAC Intervention

The intravenous dosage schedule was based on that used in acute paracetamol poisoning. The NAC administration protocol was based on the Prescott et al. protocol [29]. A continuous perfusion administration protocol of NAC was carried out with the following doses: loading dose: 150 mg/kg in 100 cc of saline to be administered over 15 min, and 50 mg/kg in 100 cc of saline solution to be administered in 4 h; maintenance dose: 50 mg/kg in 250 cc of saline to be administered at 10 cc/h for 72 h. If, after completing the 72-h infusion the patient presents PaO_2_/FiO_2_ > 200, the regimen was changed to 600 mg IV every 12 h. In the event that the patient continues with PaO_2_/FiO_2_ <200, the infusion was maintained until this target was achieved and then adjusted to a 600 mg IV every 12 h.

### 2.4. Data Collection

On the day of ICU admission and on the third day, the following data were recorded: patient age, sex, Acute Physiology and Chronic Health Assessment II (APACHE II) score [30], Sequential Assessment of Organ Failure (SOFA) score [31], duration of ICU stay, days of mechanical ventilation, patient mortality at 28 days and cardiocirculatory parameters (mean blood pressure, heart rate, blood pressure, respiratory rate, and other respiratory function variables such as FiO_2_ and PaO_2_/FiO_2_ were also obtained). To calculate the days of mechanical ventilation and the stay in the ICU, patients who survived were considered.

### 2.5. Biochemical Parameters

Initial and final plasma and erythrocyte samples were collected under fasting conditions, followed by centrifugation (4 °C for 15 min at 3500 rpm) to separate plasma and serum. The samples were stored at −80 °C before biochemical analysis for subsequent tests. Plasma and erythrocyte samples were obtained from the NAC-treated and control group patients. The following initial and final data were recorded: biochemical blood profile acid-base balance: pH; renal function: creatinine, urea, and ions; liver function: glutamic oxaloacetic transaminase (GOT) and glutamic pyruvic transaminase (GPT); haematometric parameters: leukocytes, neutrophils, lymphocytes, and rate neutrophils/lymphocytes; inflammatory parameters: lactate dehydrogenase (LDH), PCR, lactate, ferritin, D-dimer, and procalcitonin.

### 2.6. Assessment of GSH and GSSG

A colorimetric detection kit (Invitrogen by Thermofisher Scientific, ref: EIAGSHC, Madrid, Spain) was used to perform the GSH and GSSG determination assay. The erythrocyte samples were treated with sulfosalicylic acid to precipitate the proteins. Thereafter, 0.050 mL of the sample was placed in an Eppendorf tube and 0.150 mL of 5% sulfosalicylic acid was added. Preparation of sulfosalicylic acid: 1 g of sulfosalicylic acid was placed in a beaker and made up to 20 mL with distilled water. Samples were shaken and then incubated at room temperature for 10 min, then centrifuged at 14,000 rpm for 10 min at 4 °C. The supernatant was diluted with 1% sulfosalicylic acid. Then, 0.05 mL of the diluted sample was transferred to the test well together with a colorimetric detection reagent, glutathione reductase, and NADPH. The absorbance was measured at 405 nm in a microplate reader (Biostack neo. BiotTek. By Izasa Scientific, Madrid, Spain). An assay curve was also constructed and measured, which was then used to extrapolate the absorbance and obtain the concentration of the samples. To measure oxidized glutathione, the same procedure was followed by adding 2-vinylpyridine to the sample. Two quality controls from two known concentrations of the calibration curve were used. Samples from a temperature of −80 °C were kept cold and under the same conditions throughout the determination process by a researcher specialized in clinical analysis.

### 2.7. Assessment of Erythrocyte Glutathione Peroxidase Activity (GPx1 Activity)

The GPx1 activity of red blood cell hemolysate was assessed with a colorimetric assay using the Bioxytech^®^ kit (OxisResearch™, ref: IMKPA071026E, Shizuoka, Japan). Aliquots of erythrocytes were mixed into the four volumes of distilled water and centrifuged at 10,000 rpm for 15 min at 4 °C, followed by the addition of 3× Assay Buffer. The sample was added to the test well along with the reagents (NADPH and tert-Butyl Hydroperoxide) and the absorbance was measured in a microplate reader every 30 s for 3 min (Biostack neo. BiotTek. By Izasa Scientific, Madrid, Spain). Enzyme activity was evaluated at 25 °C at a wavelength of 340 nm.

### 2.8. Statistical Analysis

Statistical analysis was performed with SPSS version 21.0 for Windows (SPSS Inc., Chicago, IL, USA). Qualitative variables were presented as frequencies and percentages of patients, and quantitative variables as mean ± standard deviation (SD). For continuous variables, the assumption of normality was tested using the Shapiro–Wilk test. The differences in biochemical parameters and clinical outcomes between treated and control group patients were evaluated by Student’s *t*-test for parametric samples. The chi-square test was used to assess the differences between treated and control group patients for qualitative variables. The evolution of the critically ill patients with COVID-19 in the ICU (first and third day of admission) was evaluated by the paired Student’s *t*-test for parametric samples and the Wilcoxon test for non-parametric variables. Correlations between biochemical parameters and clinical outcomes were determined using Pearson’s correlation coefficient for parametric variables and Spearman’s correlation coefficient for non-parametric variables. Statistical significance was set as *p ≤* 0.05.

## 3. Results

### 3.1. Patient Characteristics

Table 1 shows the clinical variables and the differences between treated and control group patients. Gender-based differences were observed in ICU admission in patients affected by COVID-19, being more frequent in men than in women (chi-square = 38.3; *p ≤* 0.001). Of the 140 patients, 57.7% were non-smokers, 34.5% were ex-smokers, and 7.8% were smokers. Most patients diagnosed with COVID-19 presented dry cough, fever, asthenia, myalgia, ageusia, and anosmia.

Most patients had underlying diseases such as cardiovascular diseases, hyperlipidemia, diabetes, and chronic obstructive pulmonary disease. The mean (SD) Acute Physiology and Chronic Health Assessment II (APACHE II) and Sequential Assessment of Organ Failure (SOFA) scores at admission were 14.5 (8.6) and 2.4 (1.7), respectively. No differences in SOFA scores were found throughout the ICU stay in either NAC-treated or control groups. Mechanical ventilation was required for 79.3% of patients (these patients received vasoactive support), whereas 29.3% required only a high-flow nasal cannula (HFNC). The mean (SD) length of ICU stay was 24.3 (22.7) days, and the mean days under mechanical ventilation were 22.9 days in all patients (20.4). The observed 28-day mortality was 37.9% (53 patients). Clinical characteristics of the NAC-treated and control group patients were similar on the first day of ICU admission.

### 3.2. Biochemical Parameters

Table 2 represents the comparative clinical characteristics, GSH and GSSG activities, and erythrocyte GPx activity at admission and on the third day of ICU stay in COVID-19 NAC-treated and control group patients. All parameters were altered, with very high levels of acute markers of inflammation, such as CRP, ferritin, and D-dimer, together with kidney and liver failure markers (all, *p* < 0.042). Regarding plasma glutathione and GPx activity, no differences were found in NAC-treated or control group patients between the first and third days of ICU stay. Both groups showed similar behavior regarding glutathione changes in erythrocytes. In the NAC-treated and control group patients, a decrease in the glutathione concentration was found on the third day compared with the first day of ICU stay.

### 3.3. Association of Mortality with GSH, GSSG, and GPx

Table 3 shows the comparative levels of GSH, GSSG, and GPx with mortality at 28 days in the NAC-treated and control group patients with COVID-19. It was observed that total GSH levels at admission were significantly higher (*p ≤* 0.041) in those patients who died than in survivors in NAC-treated patients and close to statistical significance (*p ≤* 0.069) in control group patients. No significant differences in glutathione (erythrocyte) concentration were found between the survivors and the deceased patients on the third day of ICU stay.

### 3.4. Association between GSH, GSSG, and GPx with Clinical Outcomes and Severity Biomarkers

Table 4 reports the association between GSH, GSSG, and GPx and clinical outcomes and severity biomarkers in the NAC-treated and control group patients with COVID-19. Table 4 shows that more correlations between glutathione and inflammatory parameters were found in NAC-treated patients than in control group patients, for whom no correlations were found. In NAC-treated patients, positive correlations between glutathione and severity parameters such as SOFA or lactic acid (r = 0.262 to 0.693; *p ≤* 0.01) were found on the first day and third days of ICU stay. In NAC-treated patients, positive correlations were found with renal parameters such as creatinine or urea (r = 0.287 to 0.611; *p ≤* 0.05 to *p ≤* 0.01) on the first and third days of ICU stay. In the case of sodium, a positive correlation was found with the GSH/GSSG (erythrocyte) rate (r = 0.373; *p ≤* 0.01) on the third day, which was not found in the control group patients. Moreover, negative correlations were found between the inflammatory parameters, that is, fibrinogen (r = −0.266; *p ≤* 0.01) and ferritin (r = −0.245; *p ≤* 0.05) and GSSG; and positive correlations were found between hematologic parameters, that is, leukocytes with total GSH (r = 0.332; *p ≤* 0.05) and GSSG (r = 0.287; *p ≤* 0.01); and between Neutrophils/Lymphocytes ratio and total GSH (r = 0.295; *p ≤* 0.05) and GSSG (r = 0.332; *p* ≤ 0.05); also negative correlations were found between hemoglobin and total GSH (r = −0.296; *p ≤* 0.05) and GSSG (r = −0.333; *p ≤* 0.05) in NAC-treated patients on the third day of ICU stay; these correlations were absent in the control group on the third day. Regarding neutrophils and lymphocytes, correlations with glutathione were found in both the NAC-treated and control group patients, but in the case of NAC-treated correlations were only found on the third day and were positive in neutrophils (r = 0.290 to 0.377; *p ≤* 0.05 to *p ≤* 0.01) and negative in lymphocytes (r = −0.278 to −0.355; *p ≤* 0.05 to *p ≤* 0.01).

## 4. Discussion

The main results of the present study revealed that in NAC-treated patients, PaO_2_/FiO_2_ increased on the third day compared to those control group patients, in whom no changes were observed during the 3 days of stay in the ICU. Moreover, NAC also managed to decrease CRP, D-dimer, and LDH levels in patients treated with NAC, with a smaller decrease in total GSH being observed in NAC-treated patients than in the control group. This is the first study to address the glutathione response to NAC administration, as other similar studies only compare clinical and biochemical outcomes in NAC-treated and control group patients with COVID-19 [23,24,32,33]. Finally, associations between glutathione and clinical outcomes and severity biomarkers were found in NAC-treated patients, which were not found in control group patients, which may justify the effect that the administration of NAC had on the patient’s ICU stay.

In our study, both the NAC-treated and control group patients had an altered clinical outcome on the first day of the study. Moreover, a decrease in the positive end-expiratory pressure (PEEP) and an increase in PaO_2_/FiO_2_ was found in our NAC-treated patients on the third day, whereas no changes in this regard were found in the control group. In this line, a previous study showed a clinical improvement in patients treated with NAC, with a similar increase in PaO_2_/FiO_2_ [32].

The CRP, D-dimer, and LDH responses to IV NAC were favorable in our patients. It should be noted that the decrease in D-dimer should be interpreted with caution since D-dimer levels on the initial day were significantly higher in the treated patients than in the control group. In particular, it can be seen that the D-dimer results showed high intragroup variability, therefore, quantitative difference between the first and the third day was calculated, observing statistically significant differences in the evolution in ICU (*p* =0.009). In this regard, the decrease obtained in the treated group on the third day may be due to the administration of NAC. Patients with COVID-19 can present blood coagulation abnormalities, primarily manifested by elevated levels of fibrinogen and D-dimer in tandem with mild thrombocytopenia [34,35]. D-dimer levels have been associated with a worse prognosis of morbidity and mortality [36,37]. D-dimer levels, lung inflammation, and pulmonary hemorrhage are influenced by neutrophil elastase activity [38,39]. Therefore, suppression of elastase and neutrophil activation may be helpful in hemorrhagic or thrombotic complications associated with COVID-19 [40]. High concentrations of NAC have been found to inhibit elastase release and modulate neutrophil activity [41]. In neutrophilic airway inflammation in cystic fibrosis, high-dose NAC decreases the neutrophil burden in airways and the number of airway neutrophils actively releasing elastase-rich granules [42]. NAC can also ameliorate elastase-induced pulmonary emphysema, as shown by improved airspace expansions, partial recovery of expiratory flows, and normalization of lung collagen content [43]. All this supports the usefulness of NAC in mediating inflammation-mediated lung injury and blood coagulation abnormalities in severe cases of COVID-19.

The antiviral [44,45] and anti-inflammatory [46,47,48] properties of NAC have been previously reported. On the one hand, elevated levels of proinflammatory cytokines have been identified in the serum of patients with COVID-19 [49,50]. Specifically, interleukin-6 (IL-6) has been proposed to play an essential role in COVID-19-associated cytokine storms [51]. In this respect, NAC has been found to reduce IL-6-dependent CRP elevation during H1N1 influenza pneumonia [52]. On the other hand, preclinical studies have shown that GSH-capped nanoclusters inhibit coronavirus replication through blockage of viral RNA synthesis and budding [20]. Furthermore, an in vitro study showed that NAC was able to reduce H5N1 viral replication [45]. Moreover, the post-translational disulfide bond between the two cysteine residues (C156 and C167) is apparently essential for fusion complex exposure and the subsequent membrane fusion [53], which may be disrupted by NAC. Moreover, NAC blocks mTOR [46] which is a central regulator of inflammation within the immune system [54] and is required for the binding of its substrates LARP1 and FKBP7 to viral N and ORF8 proteins [55]. Moreover, a decreased acidity was found in NAC-treated patients after 3 days of ICU stay. This increase in pH by NAC may be due to the decrease in pyroglutamic acid levels that are high in critically ill patients due to glutathione depletion [56]. When glutathione levels are restored thanks to NAC, pyroglutamic levels can decrease, and acidemia decreases. On the third day of ICU stay, NAC-treated patients showed a decrease in CRP levels and although this decrease was also found in the control group it was more significant in the NAC-treated patients. In addition, D-dimer increased in the control group patients, however, a decrease in LDH in the control group patients was not observed. In this sense, the decrease in these three molecules after NAC treatment has been previously reported [32].

In our study, both groups of patients showed a decrease in total glutathione levels on the third day. This decrease could have been due to glutathione consumption because of elevated oxidative stress during the ICU stay, as previously described in the literature [57], although this was not assessed in the present study. The fact that the decrease in GSH on the third day was significant in the NAC-treated group and not significant in the control group could be due to the observed difference in GSH concentration between the NAC-treated group and the control group on the first day of the study, with GSH being higher in the control group. Moreover, it can be observed that the control group decreases its concentrations by half; however, this decrease is not statistically significant. We attribute these results to the large intra-group variability. Moreover, there are differences in the concentration of the total glutathione molecule on the third day of study between the NAC-treated patients and the control group, which means that NAC manages to reduce to a lesser degree the glutathione molecule, that is, it slows down the consumption of the glutathione molecule thanks to the availability of the amino acid cysteine for the de novo synthesis of the glutathione molecule. An association between mortality and glutathione levels was found in both the NAC-treated and control group patients, so glutathione levels were higher in deceased patients than in the survivors, mainly on admission. The latter could be attributed to the higher demand for glutathione occurring in the most seriously ill patients due to the generation of a larger number of free radicals. The scientific literature is controversial regarding the response of NAC administration in different pathological situations. On the one hand, several studies have shown no effect of NAC administration on glutathione concentrations in patients with schizophrenia [58], chronic hepatitis C [59], and diabetes mellitus [60,61]. On the other hand, several studies have reported that NAC could increase glutathione concentrations or the GSH/GSSG ratio in patients with adult ARDS [62,63], cystic fibrosis [42], idiopathic pulmonary fibrosis [64], fibrosing alveolitis [65], tuberculosis or HIV [66,67], and mild chronic obstructive pulmonary disease [68,69]. Some studies also showed that NAC administration increases GPx activity in patients with rheumatoid arthritis [70].

Our study found a larger association between glutathione levels and clinical outcomes such as SOFA and inflammatory parameters in NAC-treated patients and none in the control group patients on the first and third days of ICU stay. It has been suggested that NAC is not an antioxidant molecule itself but that its actual role lies in the specific replenishment of GSH in deficient cells, and NAC is likely to be ineffective in GSH-replete cells [71]. The latter leads us to interpret that NAC levels could have helped improve the parameters of patients with glutathione deficiency in their cells, who had the worst prognosis.

Concerning clinical outcomes, previous studies in NAC-treated patients infected by SARS-CoV-2 showed no decreased intubation rate, no improvement in oxygenation index, no shortening of ICU stay, nor reduction in mortality [23,24]. Moreover, a double-blind, randomized study with a placebo and with a NAC regimen similar to ours in 140 severely ill patients with COVID-19 found no differences between cases and controls regarding the time of mechanical ventilation, the time in ICU, and the mortality [23]. Furthermore, a study involving 92 patients divided into NAC-treated and control group patients reported no differences in the mortality rate at 28 days, finding similarities between groups and the proportion of patients who required invasive ventilatory support (38.3% vs. 44.4%, respectively), number of days without mechanical ventilation (17.4 vs. 16.6, respectively), and median length of stay in the ICU and hospital. The results regarding the change in the PaO_2_/FiO_2_ ratio and SOFA scores also showed no significant differences between the groups [24].

However, oral administration of NAC (1200 mg/day) in patients with COVID-19 pneumonia decreases the risk of mechanical ventilation and mortality [32]. Eighty-two patients enrolled in the study (42 in the NAC group and 40 in the control group), and the treatment with oral NAC led to significantly lower progression rates to severe respiratory failure. Furthermore, those NAC-treated patients had lower mortality at 14 and 28 days than controls, decreasing 14-day and 28-day mortality in patients with severe disease. In addition, NAC improved the PaO_2_/FiO_2_ ratio over time, in consistency with our study, and decreased the levels of white blood cells, CRP, D-dimers, and LDH. Another NAC-intervention study revealed, in the group of NAC-treated patients compared to the control group patients, increases in blood oxygen saturation and oxygenation index, a difference in delta increase in oxygenation index, a more rapid decrease in the volume of lung damage, in the delta reduction of this index, a decrease in CRP (as in our study), and hospital stay length [33]. In another study conducted on NAC-treated patients with ARDS, an improvement was found compared with the control group (placebo), increasing PaO_2_/FiO_2_ (as in our study) and decreasing the mortality rate [72]. A decrease in comorbidity and mortality was also demonstrated in patients with severe COVID-19 after administering a NAC derivative [73].

Despite the results of the present study, this work is not without limitations. Firstly, data on patients with mild symptoms were not available because the samples were collected during the highest peak of the pandemic. Secondly, the recruited patients were from a single hospital and some potential confounding factors (sociodemographic and socioeconomic status) were not evaluated. Thus, these outcomes cannot be generalized to other populations, especially considering the wide range of COVID-19 prevalence. Thirdly, the methodology used in the determination of the glutathione molecule, despite a validated colorimetric method, may not achieve sufficient sensitivity to determine this molecule, and a chromatographic method such as HPLC may be more appropriate. Finally, the overall results may be related to the heterogeneity of the subjects and their underlying disease conditions or severity.

## 5. Conclusions

The administration of NAC manages to improve the clinical and analytical response of seriously ill patients with COVID-19 compared to the control group. NAC is able to stop the decrease in glutathione concentrations. Therefore, the administration of NAC in critically ill patients with COVID-19 could be assessed based on the need for quick and agile intervention through monitoring and follow-up in the ICU from the beginning of the stay to prevent and correct possible alterations and improve prognosis.

## Figures and Tables

**Table 1:** Clinical characteristics and differences between treated and control group critically ill patients with COVID-19 in the initial day.

	Treated Patients (*n =* 72)	Control Group Patients (*n =* 68)	*p*-Value (Treated vs. Control Group)
Age, (years)	61.4 (12.3)	62.2 (10.2)	0.696
Male, number (%)	56 (78.9%)	50 (73.5%)	0.294
ICU stay (days)	26.2 (25.5)	22.1 (19.1)	0.403
Mechanic ventilation (days)	24.6 (23.1)	20.7 (16.2)	0.460
Mechanic ventilation, number (%)	60 (84.5%)	50 (73.5%)	0.083
SOFA score	4.51 (1.96)	5.01 (2.57)	0.197
APACHE II score	13.5 (5.8)	17.5 (13.9)	0.262
Mortality, number (%)	25 (35.2%)	28 (41.2%)	0.291
MBP (mmHg)	98.9 (16.3)	96.2 (16.5)	0.153
PaO_2_/FiO_2_	168.6 (74.9)	179.0 (73.1)	0.478

Values are expressed as mean ± standard deviation; the fourth column shows the statistical significance after applying the tests to discern if there are differences between treated and control group patients. SOFA score: Sequential Assessment of Organ Failure. APACHE II: score Acute Physiology and Chronic Health Assessment II. MBP: Mean Blood Pressure. PaO_2_/FiO_2_: Partial Oxygen Arterial Pressure/Fraction of Inspired Oxygen.

**Table 2:** Comparative clinical characteristics, severity biomarkers, GSH and GSSG activities, and erythrocyte GPx activity at admission and at three days ICU stay in COVID-19 patients treated and control group with NAC.

	Control Group Patients		*p*-Value (Initial vs. Final)	Treated Patients		*p*-Value (Initial vs. Final)	*p*-Value (Treated vs. Control Group) Initial	*p*-Value (Treated vs. Control Group) Final
	Initial	Final		Initial	Final			
SOFA score	4.51 (1.96)	4.74 (2.79)	0.425	5.01 (2.57)	4.89 (2.62)	0.554	0.197	0.758
HR (bpm)	76.5 (16.6)	67.0 (17.1)	0.001	80.2 (20.3)	68.6 (18.5)	0.001	0.313	0.377
BF (bpm)	26.0 (6.2)	21.8 (3.7)	0.001	26.8 (6.3)	22.0 (5.6)	0.001	0.008	0.714
MBP (mm Hg)	98.9 (16.3)	86.9 (13.2)	0.001	96.2 (16.5)	89.3 (14.9)	0.566	0.153	0.364
PEEP (cm H_2_O)	11.8 (2.7)	11.9 (1.7)	0.858	13.4 (2.4)	12.4 (2.2)	0.001	0.002	0.101
FiO_2_ (%)	0.81 (0.19)	0.63 (0.15)	0.001	0.75 (0.18)	0.62 (0.16)	0.001	0.050	0.144
PaO_2_/FiO_2_	179.0 (73.1)	185.7 (58.3)	0.412	168.6 (74.9)	204.8 (69.1)	0.014	0.478	0.054
pH	7.37 (0.10)	7.41 (0.07)	0.179	7.34 (0.10)	7.44 (0.06)	0.001	0.018	0.323
Lactic acid (mmol/L)	1.66 (0.82)	1.36 (0.32)	0.188	1.82 (1.28)	1.68 (0.45)	0.600	0.932	0.014
Ferritin (ng/mL)	1579 (1182)	2212 (3143)	0.092	2011 (1833)	2066 (2093)	0.811	0.913	0.790
D-dimer (ng/mL)	2229 (8269)	3778 (7570)	0.044	4903 (14,616)	2786 (3702)	0.042	0.057	0.040
Creatinine (mg/dL)	1.11 (0.75)	1.07 (0.92)	0.565	1.06 (0.67)	1.02 (0.75)	0.623	0.577	0.754
Urea (mg/dL)	82.6 (49.2)	88.4 (51.6)	0.338	89.7 (60.5)	103.8 (61.1)	0.381	0.231	0.782
Sodium (mmol/L)	139.0 (4.0)	139.0 (4.3)	0.931	139.6 (4.4)	141.8 (5.3)	0.001	0.544	0.001
Potassium (mmol/L)	4.11 (0.50)	4.09 (0.55)	0.891	4.06 (0.54)	3.98 (0.49)	0.314	0.801	0.195
GOT or AST (U/L)	42.4 (28.2)	109.7 (590.8)	0.354	50.9 (53.4)	35.1 (22.8)	0.016	0.290	0.320
GPT or ALT (U/L)	43.3 (34.9)	72 (163)	0.147	61.2 (84.2)	63.5 (73.0)	0.749	0.113	0.740
CRP (mg/L)	114.6 (78.5)	93.8 (92.9)	0.023	131.3 (93.0)	71.4 (68.0)	0.001	0.266	0.108
Procalcitonin (ng/dL)	0.33 (0.52)	1.00 (6.15)	0.401	0.51 (1.32)	0.26 (0.46)	0.164	0.298	0.284
LDH (U/L)	544.8 (187.9)	584.4 (800.8)	0.686	546.5 (220.6)	456.0 (135.3)	0.001	0.682	0.450
Leukocytes (∗10^3^/µL)	11.96 (5.75)	11.35 (5.69)	0.328	11.32 (5.29)	10.52 (4.48)	0.176	0.380	0.348
Neutrophils (∗10^3^/µL)	10.74 (5.42)	9.78 (5.40)	0.116	9.59 (4.67)	8.94 (4.23)	0.233	0.137	0.294
Lymphocytes (∗10^3^/µL)	0.71 (0.34)	0.91 (0.48)	0.001	0.70 (0.44)	0.93 (0.92)	0.039	0.551	0.959
Rate N/L Total	18.1 (10.5)	15.4 (18.2)	0.194	18.6 (14.5)	17.0 (22.0)	0.439	0.674	0.642
Hemoglobin (g/dL)	13.7 (2.0)	12.7 (1.8)	0.001	13.0 (2.1)	12.2 (2.3)	0.001	0.103	0.147
GSH (µM) (plasma)	6.03 (6.66)	6.71 (3.46)	0.786	4.12 (1.80)	4.74 (2.05)	0.407	0.155	0.377
GSSG (µM) (plasma)	1.89 (1.64)	2.50 (2.13)	0.489	1.15 (0.92)	1.67 (1.33)	0.265	0.108	0.790
GSH/GSSG (plasma)	10.33 (18.77)	5.52 (4.05)	0.479	7.01 (10.27)	5.64 (8.02)	0.717	0.572	0.831
GSH (µM) (erythrocyte)	224 (227)	119 (121)	0.191	159 (151)	117 (109)	0.041	0.022	0.893
GSSG (µM) (erythrocyte)	109 (66)	63 (52)	0.010	119 (83)	104 (73)	0.039	0.451	0.001
GSH/GSSG (erythrocyte)	1.72 (1.30)	1.84 (1.40)	0.857	2.02 (3.45)	1.13 (0.64)	0.100	0.064	0.001
Total GSH (µM)	467 (311)	251 (186)	0.047	404 (265)	325 (241)	0.004	0.311	0.057
GPx1 (mU/mL) (erythrocyte)	2797 (1143)	3172 (1729)	0.368	2978 (700)	2925 (687)	0.592	0.224	0.211

Values are expressed as mean ± standard deviation; the fourth and seventh columns show the statistical significance after applying the comparison of means for related samples, thus, the evolution of PaO_2_/FiO_2_ is shown after three days. The eighth and ninth columns show the comparison of means for independent samples between cases and controls. SOFA score: Sequential Assessment of Organ Failure. ER: Heart rate. MBP: BF: Breathing frequency. Mean Arterial Blood Pressure. PEEP: positive end-expiratory pressure. FiO_2_: Fraction of Inspired Oxygen.: Partial Oxygen Arterial Pressure/Fraction of Inspired Oxygen. GOT or AST: glutamic oxaloacetic transaminase or aspartate transaminase. GPT or ALT: glutamic pyruvic transaminase or alanine transaminase. CRP: C-reactive protein. LDH: lactate dehydrogenase. Rate N/L: Rate Neutrophils/Lymphocytes. GSH: reduced glutathione. GSSG: oxidized glutathione. GSH/GSSG: reduced glutathione/ oxidized glutathione. GPx1: glutathione peroxidase activity.

**Table 3:** Comparative levels of GSH, GSSG, and GPx and 28-day mortality in the NAC-treated and control group patients with COVID-19.

	28-Day Mortality First Day			28-Day Mortality Third Day		
	Survivors (Mean ± SD)	Deceased (Mean ± SD)	*p*-Value	Survivors (Mean ± SD)	Deceased (Mean ± SD)	*p*-Value
Control group patients						
GSH (µM) (erythrocyte)	196.5 (192.1)	322.7 (295.3)	0.184	105.4 (171.6)	119.5 (132.2)	0.770
GSSG (µM) (erythrocyte)	88.1 (70.6)	145.5 (71.0)	0.034	65.2 (68.2)	51.4 (39.6)	0.441
GSH/GSSG (erythrocyte)	3.24 (4.04)	3.11 (2.95)	0.936	2.30 (2.10)	2.82 (1.48)	0.383
Total GSH (µM)	372.4 (304.6)	630.4 (361.9)	0.069	224.9 (272.2)	221.9 (199.1)	0.969
NAC-treated patients						
GSH (µM) (erythrocyte)	118.6 (100.9)	200.4 (191.5)	0.040	96.3 (97.2)	134.5 (120.0)	0.216
GSSG (µM) (erythrocyte)	110.5 (81.8)	140.7 (83.7)	0.150	98.3 (77.1)	113.9 (63.9)	0.390
GSH/GSSG (erythrocyte)	1.56 (2.65)	2.19 (3.96)	0.482	0.96 (0.58)	1.08 (0.81)	0.532
Total GSH (µM)	339.7 (239.0)	486.5 (258.5)	0.041	305.4 (239.3)	353.5 (212.6)	0.476

*p* ≤ 0.05: Statistical significance. GSH: reduced glutathione. GSSG: oxidized glutathione. GSH/GSSG: reduced glutathione/ oxidized glutathione.

**Table 4:** Correlation matrix between GSH, GSSG, and GPx and clinical outcomes and severity biomarkers in the NAC-treated and control group patients.

	Control Group Patients								NAC-Treated Patients							
	GSHeri (µM) First Day	GSHeri (µM) Third Day	GSSGeri (µM) First Day	GSSGeri (µM) Third Day	GSH/GSSGeri First Day	GSH/GSSGeri Third Day	Total GSHeri (µM) First Day	Total GSHeri (µM) Third Day	GSHeri (µM) First Day	GSHeri (µM) Third Day	GSSGeri (µM) First Day	GSSGeri (µM) Third Day	GSH/GSSGeri First Day	GSH/GSSGeri Third Day	Total GSHeri (µM) First Day	Total GSHeri (µM) Third Day
SOFA score	0.216	−0.167	0.098	−0.072	0.276	0.024	0.148	−0.211	0.426 **	0.497 **	0.378 **	0.262 **	−0.005	0.375 **	0.508 **	0.392 **
Lactic acid (mmol/L)	−0.238	−0.141	0.003	−0.104	−0.196	−0.113	−0.162	−0.132	0.603 **	0.693 **	0.171	0.260	0.238	0.649 **	0.504 **	0.501 **
Fibrinogen mg/dL	−0.240	−0.175	−0.002	−0.089	−0.141	0.006	−0.176	−0.206	−0.174	0.005	−0.143	−0.266 **	−0.048	0.024	−0.247	−0.172
INR	0.170	−0.094	0.229	−0.098	−0.090	0.124	0.228	−0.106	0.062	0.298 *	0.202	0.084	−0.032	0.150	0.173	0.233
aPTT (sg)	0.372	−0.024	0.304	−0.164	−0.123	−0.029	0.423 *	−0.057	0.077	0.223	0.110	−0.025	0.077	0.272	0.164	0.115
CK U/L	0.017	−0.058	−0.001	−0.093	−0.133	−0.098	0.004	−0.053	0.062	0.070	0.331 **	0.185	−0.104	−0.065	0.248	0.158
LDH (U/L)	−0.138	−0.200	−0.003	−0.141	0.077	−0.129	−0.154	−0.160	0.036	0.217	0.159	0.186	−0.051	0.148	0.112	0.221
TnT (ng/L)	−0.197	−0.115	0.035	0.254	−0.137	−0.249	−0.033	0.118	0.209	0.177	0.281 *	−0.114	−0.061	−0.015	0.318*	0.163
CRP (mg/L)	−0.334	−0.119	0.013	−0.081	−0.281	−0.034	−0.267	−0.147	0.044	0.207	0.119	−0.090	−0.007	0.083	0.087	0.061
PCT (ng/dL)	0.101	−0.109	0.301	−0.159	−0.170	0.025	0.192	−0.145	0.119	0.217	0.034	−0.002	0.019	0.148	0.053	0.186
Ferritin (ng/mL)	−0.203	0.055	−0.005	−0.060	−0.117	−0.175	−0.144	0.043	0.024	−0.084	−0.173	−0.245 *	0.149	−0.194	−0.159	−0.217
Creatinine (ng/mL)	0.259	−0.148	0.179	−0.195	−0.105	−0.103	0.324	−0.169	0.262	0.327 *	0.287 *	0.302 *	−0.058	0.193	0.329 *	0.344 *
Urea (ng/mL)	0.409	−0.139	0.096	−0.196	0.171	−0.131	0.358	−0.172	0.425 *	0.595 **	0.206	0.464 **	0.433	0.269	0.281	0.611 **
Sodium mEq/L	0.219	0.048	−0.184	0.039	0.362	−0.121	0.076	0.077	0.033	0.212	−0.168	−0.052	0.012	0.373 **	−0.035	0.054
Proteins g/dL	0.156	−0.288	0.090	−0.053	0.280	−0.154	0.099	−0.207	−0.350 *	0.067	−0.030	−0.079	−0.298 *	0.150	−0.203	−0.044
Leukocytes ∗10^3^/µL	−0.215	0.206	0.013	0.105	0.041	0.067	−0.152	0.158	−0.058	0.153	0.042	0.332 **	−0.046	0.072	−0.093	0.287 *
Neutrophils ∗10^3^/µL	−0.379 *	−0.234	−0.099	−0.189	−0.039	−0.334 *	−0.400 *	−0.252	−0.064	0.351 *	−0.019	0.290 *	0.044	0.130	−0.068	0.377 **
Lymphocytes ∗10^3^/µL	0.321	0.340 *	0.181	0.287	−0.071	0.300	0.351	0.382 *	−0.055	−0.349 *	0.041	−0.278 *	−0.016	−0.100	0.043	−0.355 **
N/L rate	−0.218	−0.178	−0.263	−0.197	−0.008	−0.175	−0.249	−0.211	0.160	0.262	0.111	0.295 *	−0.031	0.080	0.152	0.332 *
Hemoglobin (gr/dL)	0.055	0.014	0.208	0.002	−0.009	−0.157	0.015	0.073	0.179	−0.182	−0.120	−0.296 *	0.138	0.002	0.029	−0.333 *

* *p ≤* 0.05; ** *p ≤* 0.01= statistical significance. SOFA score: Sequential Assessment of Organ Failure. INR: International Normalized Ratio. aPTT: Partial Thromboplastin Time. CK: Creatine Kinase. LDH: lactate dehydrogenase. TnT: Troponin T. CRP: C-reactive protein. PCT: procalcitonin. N/L rate: Neutrophils/Lymphocytes rate. GSH: reduced glutathione. GSSG: oxidized glutathione. GSH/GSSG: reduced glutathione/oxidized glutathione.

## Data Availability

Data will be shared upon reasonable request by the corresponding author: Yenifer Gamarra-Morales.
